# The obesity–neuroinflammation–cancer axis: neuroimmune remodeling and therapeutic opportunities

**DOI:** 10.3389/fimmu.2026.1999371

**Published:** 2026-10-26

**Authors:** Nan Yang, Wei Zhang, Yinsheng Zhong, Zhenghua Ding

**Affiliations:** 1 Department of General Surgery, Xiangyang No 1 People’s Hospital, Hubei University of Medicine, Xiangyang, China; 2 Henan Key Laboratory of Cancer Epigenetics, Cancer Hospital, The First Affiliated Hospital, College of Clinical Medicine, Medical College of Henan University of Science and Technology, Luoyang, China

**Keywords:** cancer, immunometabolism, neuroimmune crosstalk, neuroinflammation, obesity, sympathetic nervous system, tumor microenvironment

## Abstract

Obesity is a chronic metabolic and inflammatory disorder associated with increased risk and poorer outcomes in multiple malignancies. Although adipose inflammation, insulin resistance, and altered adipokine signaling are established links between obesity and cancer, these mechanisms do not fully explain how systemic metabolic stress is translated into persistent changes in tissue immunity and tumor behavior. This narrative Review proposes the obesity–neuroinflammation–cancer axis as an integrative framework in which obesity-associated metabolic and inflammatory signals perturb central and peripheral neural homeostasis, while neuroinflammation, neural remodeling, and neuroendocrine signaling reshape the tumor microenvironment through reciprocal interactions among nerves, immune cells, stromal cells, and cancer cells. We focus on obesity-induced neuroinflammation, tumor neuroimmune remodeling, and neuroimmunometabolic feedback, highlighting sympathetic signaling, leptin–JAK/STAT3, NF-κB, inflammasome pathways, and metabolic reprogramming. We further evaluate therapeutic opportunities involving metabolic, inflammatory, and adrenergic interventions, including their combination with immune checkpoint blockade, while emphasizing inconsistent clinical evidence and the need for composite biomarkers. This framework is intended as a testable synthesis rather than a universally established causal pathway and identifies cancer-type-specific neuroimmune circuits as a priority for future translational research.

## 1 Introduction

Obesity is a major modifiable risk factor for several malignancies, including liver, esophageal adenocarcinoma, endometrial, breast, pancreatic, and colorectal cancers (1). Yet adiposity alone does not explain the heterogeneity of cancer risk or outcome. Metabolic dysfunction, chronic low-grade inflammation, adipokine imbalance, and immune remodeling provide important biological links, but they also raise a question that is less often considered: how do metabolic and inflammatory signals influence neural regulation of the tissues in which tumors develop? Obesity-associated inflammation can affect hypothalamic and other central circuits involved in metabolic homeostasis (2), while peripheral neural and neuroendocrine signals can regulate local immune and stromal responses; cancer neuroscience, meanwhile, has revealed reciprocal interactions among nerves, malignant cells, and their microenvironment (3–6).

Existing fields each capture part of this problem but none integrates all three. Obesity–inflammation research establishes systemic metabolic and immune disturbances; cancer neuroscience characterizes local nerve–tumor interactions (3–6); and neuroimmunometabolism describes the reciprocal regulation of neuronal, immune, and metabolic states (6). Bringing them together suggests that the obesity–cancer link cannot be fully explained by adipose inflammation, insulin resistance, or tumor metabolic reprogramming alone, and that neural and neuroendocrine pathways may represent an underappreciated layer of regulation between systemic metabolic stress and local tumor behavior. Under this framing, obesity-associated neuroinflammation, tumor innervation, and neuroendocrine signaling are not parallel observations but candidate components of a single feedback system whose boundaries, causality, and therapeutic implications remain to be defined.

We searched PubMed and Web of Science for English-language publications from January 2000 to August 2026 using combinations of terms related to obesity, adiposity, sarcopenic obesity, neuroinflammation, sympathetic and adrenergic signaling, autonomic regulation, cancer, the tumor immune microenvironment, myokines, and metabolism. Reference lists of relevant articles were additionally screened to identify pertinent primary studies and reviews. The retrieved literature was critically synthesized according to biological mechanisms, study design, and level of evidence, with particular emphasis on distinguishing experimental findings from clinical associations and emerging hypotheses.

## 2 From obesity to neuroinflammation and neural remodeling

Adipose tissue is an endocrine and immune organ. During obesity, adipocyte hypertrophy, hypoxia, extracellular-matrix remodeling, altered lipid handling, and immune-cell recruitment create a chronic inflammatory milieu enriched in tumor necrosis factor alpha (TNF-α), interleukin (IL)-6, IL-1β, chemokines, free fatty acids (FFAs), and damage-associated molecular patterns (DAMPs) (2, 7–9). These signals can act systemically, alter endothelial function, and influence brain–periphery communication. Adipokine imbalance adds an endocrine component: leptin generally increases with adiposity, whereas adiponectin decreases, creating conditions that can favor inflammatory signaling and metabolic dysfunction (10–12).

The brain is not insulated from systemic metabolic inflammation. Obesity-associated inflammatory mediators and altered nutrient signals can affect neurovascular interfaces and hypothalamic circuits that govern energy balance (2, 13, 14). Microglial and astrocytic responses may reinforce local inflammation, potentially feeding back on appetite, autonomic output, and metabolic regulation. This central process, however, should not be equated with the neural changes observed within a tumor. Hypothalamic inflammation and intratumoral innervation occur in different anatomical compartments; their connection is biologically plausible but requires direct evidence of altered neural output and downstream tumor effects.

The relevance of neural regulation becomes clearer when attention shifts from the brain to peripheral tissues. Cytokines, lipid mediators, and neurotrophic factors can modify the activity of neurons and nearby immune cells, while local nerves influence vascular tone, inflammation, and tissue remodeling (3–6). This bidirectional relationship offers a route by which a metabolically inflamed host environment might modify tissue responses without assuming that a uniform inflammatory signal reaches every tumor. It also directs attention to the sympathetic nervous system (SNS) and hypothalamic–pituitary–adrenal (HPA) axis, whose effects depend on exposure duration, receptor expression, and cellular context.

The sympathetic nervous system (SNS) and hypothalamic–pituitary–adrenal (HPA) axis are major effector systems connecting neural stress responses with peripheral immunity. Norepinephrine (NE) released from sympathetic terminals acts through adrenergic receptors on malignant, stromal, and immune cells, whereas glucocorticoids generated through HPA-axis activation signal through the glucocorticoid receptor (GR) (15, 16). Acute activation can be homeostatic, but chronic or spatially dysregulated signaling may favor immune suppression, altered metabolism, and tissue remodeling. Thus, duration, anatomical context, receptor expression, and cell type are as important as the presence of the signal itself.

Neuroinflammation alone is unlikely to initiate most obesity-associated cancers. A more defensible model is that it changes the tissue context in which oncogenic events occur by lowering thresholds for inflammatory amplification, immune dysfunction, and metabolic adaptation. The strength of this relationship is likely to differ among cancer types, and this heterogeneity is central to interpreting the proposed axis. The proposed sequence and its reciprocal feedback are summarized in **Figure 1**.

**Figure 1:**
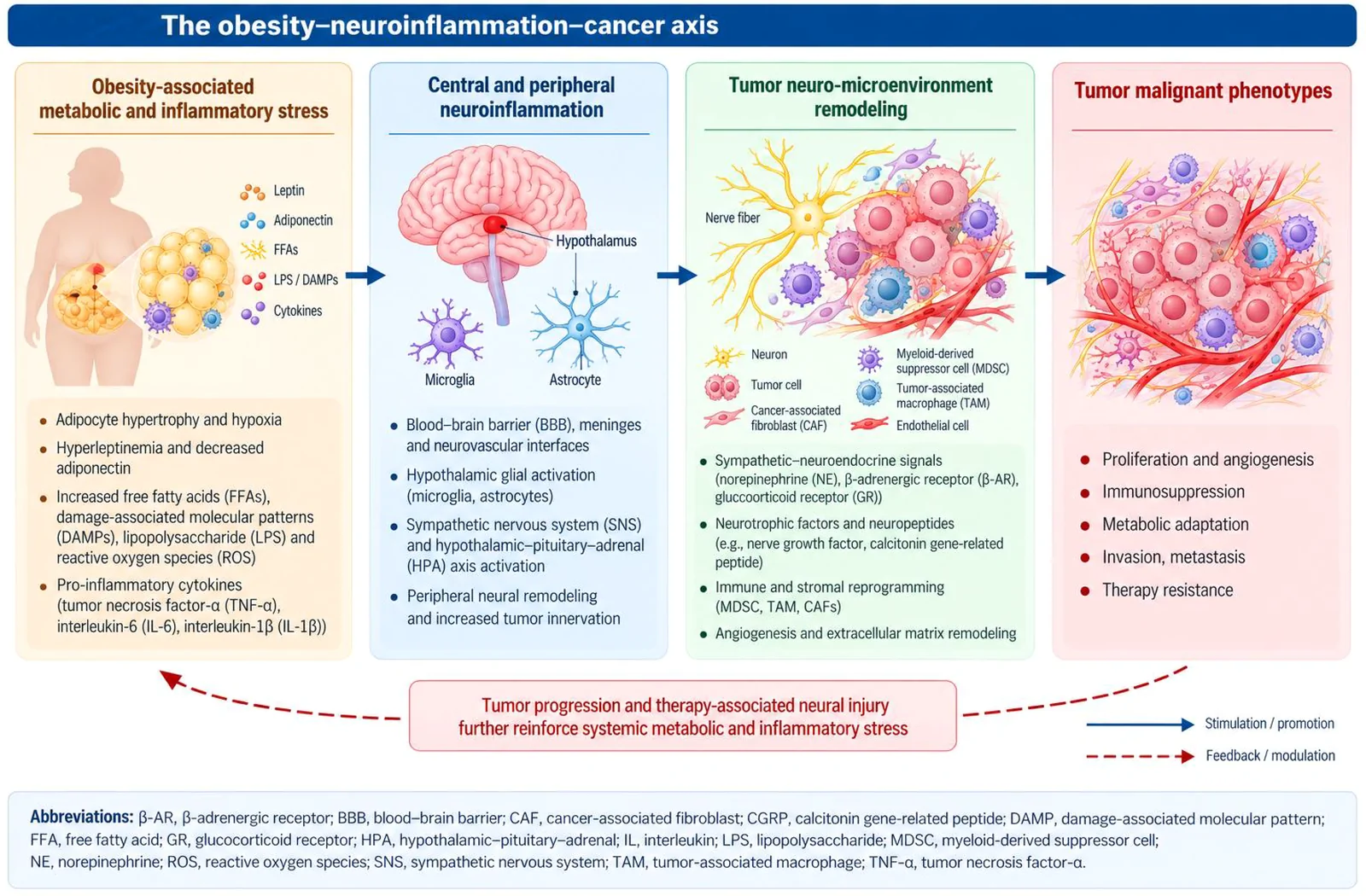
The obesity–neuroinflammation–cancer axis. Proposed integrative framework based on convergent evidence rather than a universally established linear causal pathway. Obesity-associated adipose hypertrophy, hypoxia, adipokine imbalance, free fatty acids, damage-associated molecular patterns, lipopolysaccharide, reactive oxygen species, and pro-inflammatory cytokines can perturb brain–periphery communication and promote central and peripheral neuroinflammatory responses. Neural and neuroendocrine remodeling may subsequently influence the tumor neuro-microenvironment through sympathetic signaling, neuropeptides, glucocorticoid signaling, and reciprocal interactions among malignant, immune, stromal, and endothelial cells, thereby contributing to immunosuppression, metabolic adaptation, angiogenesis, invasion, metastasis, and therapy resistance. Tumor progression and therapy-associated neural injury may feed back to reinforce inflammatory stress. β-AR, beta-adrenergic receptor; BBB, blood–brain barrier; CAF, cancer-associated fibroblast; CGRP, calcitonin gene-related peptide; DAMP, damage-associated molecular pattern; FFA, free fatty acid; GR, glucocorticoid receptor; HPA, hypothalamic–pituitary–adrenal; IL, interleukin; LPS, lipopolysaccharide; MDSC, myeloid-derived suppressor cell; NE, norepinephrine; ROS, reactive oxygen species; SNS, sympathetic nervous system; TAM, tumor-associated macrophage; TNF-α, tumor necrosis factor alpha.

### 2.1 Sarcopenic obesity as a modifier of the proposed axis

Sarcopenic obesity combines excess adiposity with reduced skeletal-muscle mass and, crucially, impaired muscle strength or function. It cannot be inferred from body mass index alone. In patients with cancer, this phenotype has been associated with poorer treatment tolerance and outcomes, although estimates vary substantially with the definitions and populations studied (17). Its relevance here is conceptual and clinically important: two patients with similar body mass index may have different inflammatory burdens, physical resilience and muscle-derived endocrine signals. Thus, adiposity without assessment of muscle quality may obscure variation in the systemic environment in which neural and tumor-immune interactions occur.

Skeletal muscle is also an endocrine organ. Exercise-responsive mediators such as irisin, the context-dependent cytokine IL-6, and the muscle-growth regulator myostatin offer plausible routes through which muscle dysfunction could modify inflammatory and metabolic signaling. IL-6 released transiently during muscle contraction should not be equated with chronically elevated IL-6 in metabolic inflammation. Likewise, evidence concerning cancer cachexia, a multifactorial wasting syndrome that can occur independently of obesity, cannot be used as direct proof about sarcopenic obesity. Whether these muscle-associated signals alter tumor innervation or neural control of antitumor immunity specifically in obese patients remains an open hypothesis, not an established mechanism (17). Recent work on cancer cachexia further details cytokine-driven muscle catabolism, but describes a syndrome distinct from sarcopenic obesity (18).

### 2.2 Cytokines and myokines at the metabolic–neural–immune interface

The relevant inflammatory signals do not form a single directional cascade from brain to tumor. Rather, adipose tissue, skeletal muscle, immune cells, neural tissues and malignant lesions can each be sources or targets, with effects determined by local receptor expression and timing. TNF-α and IL-1β participate in obesity-associated inflammatory signaling and can influence neural inflammatory responses, but their tumor effects vary with cellular context. In the tumor microenvironment, persistent inflammatory signaling may support myeloid recruitment, tissue remodeling or immunosuppressive circuits; acute inflammatory activity can have different consequences (19–21). Accordingly, elevated circulating cytokines alone do not demonstrate neural mediation of tumor progression. In glioblastoma models, IL-1β-positive tumor-associated macrophages have been linked to tumor progression through PGE2–EP4 signaling; this is tumor-specific evidence, not proof of an obesity-driven brain-to-tumor pathway (22).

TGF-β illustrates this context dependence particularly clearly: beyond its effects on stromal remodeling, it can constrain effector T-cell activity and favor regulatory immune programs in some tumors, while its roles in tumor biology vary by stage and cellular compartment (20, 21). CCL2, also known as monocyte chemoattractant protein-1 (MCP-1), is relevant because the CCL2–CCR2 axis can recruit monocytes that may differentiate into tumor-associated macrophages. This is a plausible bridge between systemic inflammation and the myeloid-rich tumor niche, but a direct obesity-to-neural-to-CCL2 sequence should not be assumed without perturbation experiments.

CX3CL1 (fractalkine) and its receptor CX3CR1 provide another example of a communication pathway shared by neural and immune compartments. The membrane-bound and soluble forms of CX3CL1 have distinct signaling implications; CX3CR1-expressing myeloid cells may respond differently across tissues and tumor types. The existence of this ligand–receptor system in neural tissues and tumors does not by itself prove that obesity-induced central neuroinflammation drives peripheral cancer through it. Myokines add a further layer of heterogeneity, because changes in muscle function can alter the systemic inflammatory milieu without necessarily changing tumor nerve density. The key experimental challenge is to identify cellular sources, receptors and temporal order rather than merely documenting co-expression (17, 21). A recent review also identifies skeletal muscle as a potential source and target of CX3CL1, connecting fractalkine signaling with muscle metabolism and local immune-cell behavior (23).

## 3 Neuroimmune remodeling in the tumor microenvironment

The tumor microenvironment brings these peripheral interactions into focus. Malignant cells can release neurotrophic factors and recruit or remodel nerve fibers, whereas neural signals may influence tumor cells, stromal elements, vascular responses, and immune surveillance (3–6). The relevance of these interactions to obesity is not that all innervated tumors are obesity-driven, but that systemic metabolic and inflammatory conditions may modify the cellular responses to local neural inputs. Accordingly, evidence from a particular tumor model should not be assumed to apply to every obesity-associated cancer. Contemporary reviews describe activity-dependent neuron–cancer interactions and tumor innervation across different malignancies (24).

Sympathetic signaling provides one experimentally tractable example. Norepinephrine can activate β-adrenergic receptors and downstream cAMP-dependent pathways in receptor-expressing malignant and stromal cells (15, 16). Immune consequences depend on which cells receive the signal: myeloid-derived suppressor cells and tumor-associated macrophages may acquire or sustain suppressive functions, while dendritic-cell antigen presentation and cytotoxic T-cell activity can be affected indirectly through changes in cytokines and the local microenvironment. Regulatory T cells add another potential layer of suppression. These outcomes are not interchangeable, and changes in immune-cell abundance alone cannot establish functional immunosuppression or altered response to checkpoint blockade.

The biological effect of these signals is determined by receptor distribution and cellular state rather than by neurotransmitter abundance alone. In myeloid populations, sustained adrenergic and inflammatory inputs can favor suppressive programs and alter cytokine production, whereas in lymphoid compartments the same stress-associated environment may impair cytotoxic effector function. Neuropeptides add further context dependence because their receptors are differentially expressed across malignant and immune-cell populations. Consequently, a tumor with dense innervation is not necessarily neuroimmune-dependent; functional receptor activity and downstream immune consequences are more informative than nerve density alone.

Neural and immune communication also runs in the opposite direction. Macrophages and other infiltrating cells release inflammatory mediators that can influence neuronal excitability, neurotrophic signaling, and local nerve remodeling. TNF-α and IL-1β illustrate how obesity-associated inflammation could alter this dialogue, whereas TGF-β may participate in stromal remodeling and immune regulation; the consequences of each mediator vary by source, receptor distribution, and cancer type (2, 7–9, 19, 20, 25). Chemokine signaling provides a more spatially specific example: CX3CL1–CX3CR1 interactions have been discussed in neural and tumor-associated inflammatory contexts, but their precise function should be established within the tissue under study rather than generalized across cancers. Thus, immune cells are not simply targets of neural regulation; they may help reshape the neural niche itself.

The consequences extend beyond primary tumor growth. Adrenergic and inflammatory signaling can influence angiogenesis, extracellular-matrix remodeling, epithelial plasticity, dissemination, and survival of residual tumor cells. Cancer therapy may add another layer: chemotherapy or radiotherapy can damage neural and stromal structures, potentially generating inflammatory signals that alter subsequent immune responses. However, the magnitude and clinical importance of treatment-associated neural injury remain incompletely defined and should not be generalized across tumor types.

Neural signals may alter antitumor immunity through several non-equivalent cellular routes. In preclinical settings, β-adrenergic input can change macrophage activation programs and myeloid-cell trafficking, whereas MDSCs suppress immunity through mechanisms that may include arginine depletion, reactive oxygen species and inhibition of T-cell function. Regulatory T cells and dendritic cells may also respond to neuroendocrine cues, but receptor expression and the direction of response are not interchangeable across models. Therefore, a mechanistic claim requires cell-specific receptor perturbation and functional assays—such as antigen presentation, T-cell killing or suppression—not simply an increase in the abundance of TAMs, MDSCs or Tregs (3, 16, 26).

It is also important to distinguish local innervation from systemic stress signaling. Norepinephrine released at sympathetic nerve terminals can act in a spatially restricted manner, whereas circulating catecholamines and glucocorticoids can influence distant tissues. Evidence from one compartment cannot establish the other. Studies combining nerve mapping, spatial immune profiling, receptor-specific perturbation and metabolic measurements would be better suited to identifying whether an obesity-associated neural state changes tumor immunity, or whether tumor-derived signals independently recruit nerves (3, 16).

## 4 Neuroimmunometabolic integration: molecular hubs and feedback loops

These interactions acquire additional significance because tumor and immune cells compete for nutrients and adapt to inflammatory stress. Obesity alters systemic glucose and lipid availability, whereas neural and neuroendocrine mediators can influence vascular function, substrate mobilization, and receptor-dependent cellular signaling (6, 27). The resulting metabolic environment may favor some immune-cell states over others. However, showing that a tumor is glycolytic or lipid-rich is not sufficient to infer that neural activity caused the phenotype; metabolic measurements need to be linked to a defined neural input.

Several molecular pathways provide points of convergence. Leptin binds the leptin receptor (ObR) and can activate Janus kinase (JAK)–signal transducer and activator of transcription 3 (STAT3), extracellular signal-regulated kinase (ERK), AKT, and c-Jun N-terminal kinase (JNK) pathways (10, 28). Nuclear factor kappa B (NF-κB) integrates inflammatory and stress signals and can support cytokine production, survival, and proliferation (19, 20). Inflammasomes, including the NOD-like receptor family pyrin domain containing 3 (NLRP3) inflammasome, connect metabolic stress and DAMPs to caspase-1 activation and maturation of IL-1β and IL-18 (25). These pathways should be viewed as interacting nodes rather than independent linear cascades.

These hubs also create mechanistic overlap between obesity biology and tumor immunology. Hyperleptinemia can provide persistent JAK–STAT3 input, while lipid-derived danger signals and oxidative stress can reinforce NF-κB and inflammasome activity. At the same time, catecholamine-responsive cAMP and PI3K–AKT/MAPK signaling can alter cellular energetics and survival programs. Rather than acting as parallel pathways, these signals may converge on myeloid-cell function, T-cell fitness, vascular remodeling, and tumor-cell nutrient use, providing a plausible basis for the self-reinforcing architecture depicted in **Figure 2**.

**Figure 2:**
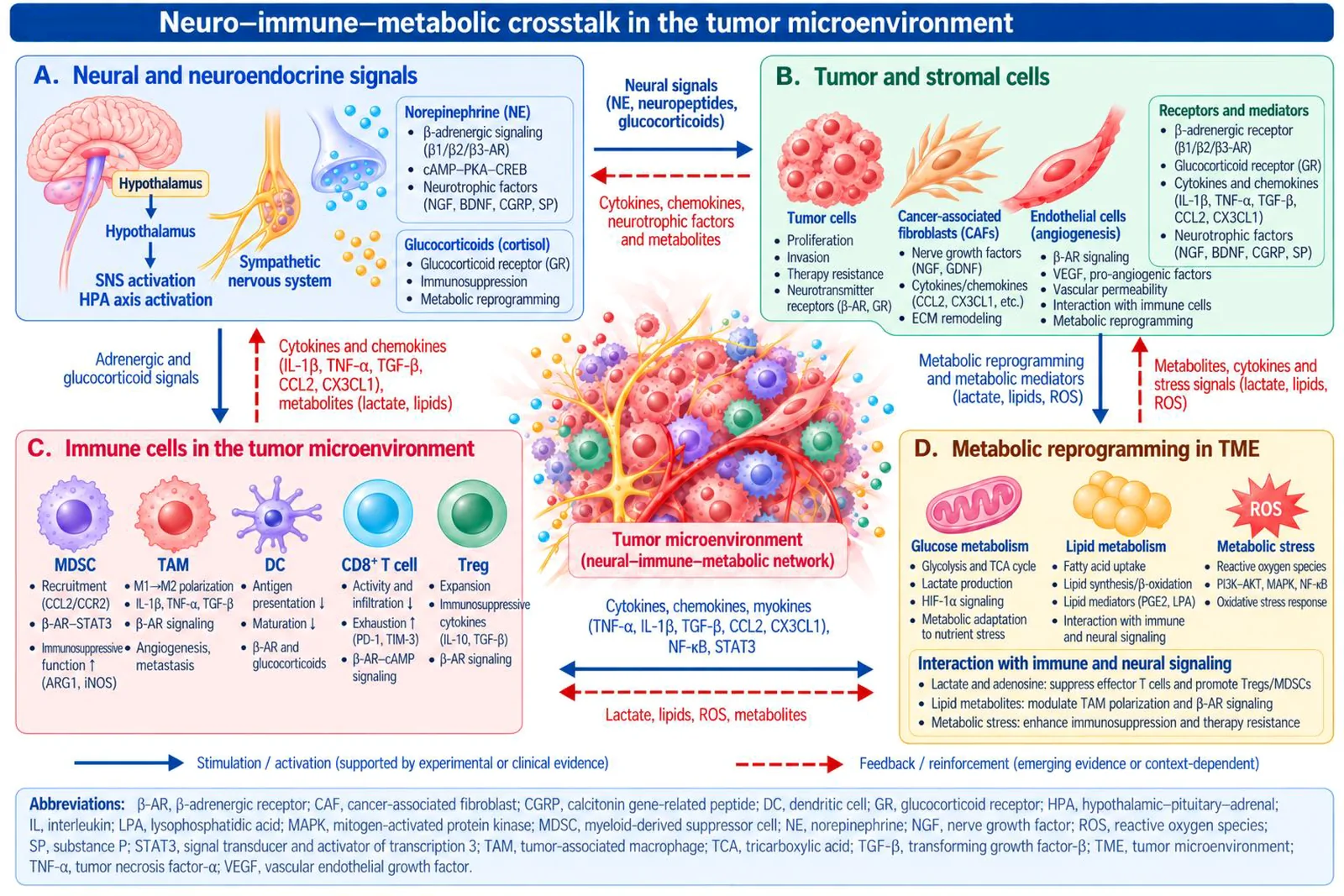
Neuro–immune–metabolic crosstalk in the tumor microenvironment. Mechanistic amplification of the tumor-microenvironment stage shown in **Figure 1**. Neural and neuroendocrine mediators, including norepinephrine, neuropeptides, and glucocorticoids, can regulate tumor, stromal, and immune compartments. Immune-derived cytokines and neurotrophic factors can reciprocally modify neural activity, while tumor and stromal cells contribute cytokines, neurotrophic factors, and metabolic mediators. Metabolic reprogramming, including glycolysis, lactate production, lipid metabolism, and reactive oxygen species, can further shape immune-cell states and inflammatory signaling. NF-κB, STAT3, PI3K–AKT, MAPK, and cAMP-linked pathways represent interacting signaling hubs rather than independent linear cascades. Solid arrows indicate stimulation or regulation; dashed arrows indicate feedback or reinforcement. β-AR, beta-adrenergic receptor; CAF, cancer-associated fibroblast; cAMP, cyclic adenosine monophosphate; CGRP, calcitonin gene-related peptide; DC, dendritic cell; GR, glucocorticoid receptor; HPA, hypothalamic–pituitary–adrenal; IL, interleukin; MAPK, mitogen-activated protein kinase; MDSC, myeloid-derived suppressor cell; NE, norepinephrine; NF-κB, nuclear factor kappa B; PI3K, phosphoinositide 3-kinase; ROS, reactive oxygen species; SNS, sympathetic nervous system; STAT3, signal transducer and activator of transcription 3; TAM, tumor-associated macrophage; TCA, tricarboxylic acid; TNF-α, tumor necrosis factor alpha.

A mechanistic connection between neural input and cancer metabolism is beginning to emerge, although it is not uniform across tumors. Adrenergic signaling can affect cellular signaling pathways that regulate nutrient use, while inflammatory mediators and adipokines can change the metabolic demands of malignant and immune cells (10, 15, 27, 28). More direct evidence comes from experimental breast cancer models demonstrating transfer of mitochondria from neurons to cancer cells during metastatic progression (29). This finding expands the range of possible neural–tumor interactions beyond neurotransmission, but does not establish that obesity-induced neuroinflammation drives mitochondrial transfer. For glucose, lipid, or lactate metabolism to be attributed to neural regulation in an obesity-associated cancer, studies must perturb the neural pathway and measure metabolic flux and downstream immune or tumor effects. A research highlight in Nature Neuroscience discusses the same mitochondrial-transfer study; the experimental claim here rests on the original Nature report (29, 30).

Obesity may intensify this loop because systemic insulin resistance, altered lipid flux, hyperleptinemia, reduced adiponectin, and chronic inflammation can pre-exist tumor development. Nevertheless, direct demonstrations that obesity-induced neuroinflammation itself causes a defined tumor metabolic program remain limited. The neuroimmunometabolic model is therefore best considered a mechanistic synthesis supported by convergent evidence rather than a universally proven linear pathway.

An important unresolved issue is the direction of causality. Obesity-associated neuroinflammation, chronic stress signaling, tumor innervation, and immune dysfunction frequently coexist, but coexistence does not establish which component is upstream in an individual cancer. Evidence is strongest for discrete links—such as obesity-associated hypothalamic inflammation, neural regulation of tumor biology, and metabolic control of immune states—whereas direct human evidence connecting the entire obesity–neuroinflammation–cancer sequence remains sparse. This distinction between established component mechanisms and inferred systems-level integration is essential when interpreting the framework and designing translational studies.

## 5 Discussion: therapeutic opportunities, biomarkers, and translational challenges

Therapeutic implications should follow the strength of the underlying evidence. Metabolic and anti-inflammatory interventions may be relevant to the host conditions described above, but plausible pathway effects do not guarantee cancer benefit. For example, the randomized MA.32 trial found no significant improvement in invasive disease-free survival with adjuvant metformin in its primary population of patients with high-risk operable breast cancer without diabetes (31). This negative result does not invalidate the underlying metabolic biology; it shows why patient selection and clinically meaningful endpoints matter (32).

Inflammatory pathways provide a second level of intervention. IL-1β, IL-6, TNF-related signaling, inflammasome activation, and myeloid-cell recruitment are attractive targets because they connect obesity-associated inflammation with immune suppression. However, clinical evidence argues against simple pathway extrapolation. In the phase III CANOPY-1 trial, adding the anti-IL-1β antibody canakinumab to pembrolizumab plus chemotherapy did not significantly improve progression-free or overall survival in advanced non-small-cell lung cancer, despite a strong inflammatory rationale (33). This negative result emphasizes that inflammatory biomarkers, tumor context, treatment timing, and pathway redundancy may determine whether anti-inflammatory combinations are effective.

Neural signaling represents a third therapeutic dimension. β-AR blockade is supported by preclinical evidence linking adrenergic stress to immune suppression, and some retrospective studies have reported improved disease control during immune checkpoint blockade (34). However, a meta-analysis of more than 10,000 patients found no significant overall-survival or progression-free-survival advantage associated with β-blocker use during immune checkpoint inhibitor (ICI) therapy (35). Similarly, clinical studies of metformin plus ICIs are inconsistent: individual retrospective cohorts have suggested benefit, whereas a 2024 meta-analysis reported no improvement and a possible adverse association with overall survival, while a later 2026 meta-analysis reported pooled benefit (36, 37). These discordant findings support prospective, stratified trials rather than empirical addition of metabolic or adrenergic drugs to immunotherapy.

The biomarker problem is therefore central to translation. A single circulating cytokine, nerve-density measurement, or receptor marker is unlikely to capture the biology of the axis. A more informative strategy would define an obesity-associated neuroimmune phenotype using complementary domains: host metabolic status and visceral adiposity; leptin/adiponectin and insulin-related measures; systemic inflammatory mediators; intratumoral neural density or activity; β-AR and GR signaling; MDSC, TAM, and cytotoxic T-cell states; and tumor metabolic features. Such composite signatures could identify patients in whom neural or inflammatory signaling is not merely present but functionally linked to immune dysfunction and treatment response.

Combination strategies should therefore be mechanism- and biomarker-guided. Potential approaches include metabolic intervention plus ICI therapy, adrenergic modulation plus ICI therapy, or selective inflammatory blockade in tumors with demonstrable inflammatory dependence. Positive observational signals can justify prospective testing, but negative randomized evidence such as CANOPY-1 and inconsistent metformin/β-blocker data show that biological plausibility alone is insufficient. Safety also matters because neural and inflammatory pathways have essential physiological functions; systemic blockade can produce cardiovascular, metabolic, neurological, or infectious consequences. The principal components of the proposed axis, their interpretive limitations, and corresponding therapeutic implications are summarized in **Table 1**.

**Table 1:** Key components of the obesity–neuroinflammation–cancer axis.

Component	Principal target/context	Representative signaling	Potential cancer relevance	Interpretive caution	Therapeutic implication
Obesity-associated inflammation	Brain interfaces, glia, peripheral neural microenvironment	IL-6, TNF-α, IL-1β, FFAs; barrier dysfunction	Chronic inflammatory background and altered neural homeostasis	Direct tumor causality is cancer-type dependent	Metabolic control and selective anti-inflammatory strategies; requires phenotype selection
Leptin excess/resistance	Neurons, immune cells, tumor cells	ObR–JAK/STAT3; ERK; AKT	Inflammatory signaling, survival, proliferation, angiogenic programs	Effects vary with receptor expression and tumor context	Potential biomarker/target within an adipokine-defined subgroup; not a stand-alone universal target
Sympathetic activation	Tumor cells, MDSCs, macrophages, T cells	NE–β-AR; cAMP; PI3K–AKT/MAPK	Immune suppression, metabolic adaptation, invasion and metastasis	Clinical β-blocker data with ICIs are inconsistent	Prospective testing of adrenergic modulation with immune therapy in β-AR-active tumors
Inflammasome activation	Glia and peripheral immune cells	NLRP3–caspase-1–IL-1β/IL-18	Inflammatory amplification and immune remodeling	Inflammasome effects can be tumor-promoting or context dependent	Selective inflammasome/IL-1 pathway inhibition; biomarker selection needed
HPA-axis signaling	Immune and tumor cells	Glucocorticoid–GR signaling; NF-κB/AP-1 modulation	Altered immune surveillance and stress adaptation	Acute and chronic effects differ	Avoid indiscriminate blockade; consider GR activity and treatment-related glucocorticoid exposure
Tumor-associated neural remodeling	Sensory and autonomic nerves	Neurotrophic and neurotransmitter signaling	Growth, angiogenesis, immune remodeling, dissemination	Bidirectional and strongly cancer-type specific	Target nerve–tumor signaling only where functional dependence is demonstrated
Neuroimmune–metabolic feedback	Cancer cells, myeloid cells, lymphocytes	Glycolysis, lipid/glutamine metabolism; cytokine feedback	Metabolic adaptation, immune dysfunction, treatment resistance	Integrative concept with limited causal human evidence	Composite biomarker-guided combinations linking metabolic, neural, and immune interventions

β-AR, beta-adrenergic receptor; AKT, protein kinase B; AP-1, activator protein 1; cAMP, cyclic adenosine monophosphate; ERK, extracellular signal-regulated kinase; FFA, free fatty acid; GR, glucocorticoid receptor; HPA, hypothalamic–pituitary–adrenal; ICI, immune checkpoint inhibitor; IL, interleukin; JAK, Janus kinase; MAPK, mitogen-activated protein kinase; MDSC, myeloid-derived suppressor cell; NE, norepinephrine; NF-κB, nuclear factor kappa B; NLRP3, NOD-like receptor family pyrin domain containing 3; ObR, leptin receptor; PI3K, phosphoinositide 3-kinase; STAT3, signal transducer and activator of transcription 3; TAM, tumor-associated macrophage; TNF-α, tumor necrosis factor alpha.

Several methodological challenges remain. Obesity, neural activity, immunity, and cancer are often studied at different biological scales, making causal inference difficult. Sympathetic, parasympathetic, and sensory neurons are heterogeneous; immune-cell states cannot be reduced to simple binary classifications; and tumor type, stage, treatment exposure, and host metabolic phenotype can change the direction of an observed effect. Future studies should integrate metabolic phenotyping with spatial transcriptomics, single-cell sequencing, multiplex imaging, neural tracing or functional imaging, immune profiling, and longitudinal clinical sampling. Particularly valuable will be studies that perturb defined neural circuits while measuring immune and metabolic consequences in obesity-relevant tumor models, followed by validation in human cohorts.

### 5.1 Cancer-specific evidence and limits of generalization

The proposed framework should not be interpreted as equally supported in every obesity-associated malignancy. The strength of evidence linking excess adiposity to cancer risk is not the same as the strength of evidence for a neural mechanism in that cancer, and neither establishes a successful neural-targeted therapy. Breast, colorectal, pancreatic, endometrial, esophageal and liver cancers also differ in tissue innervation, local immune ecology, hormonal regulation and metabolic exposures. Prostate and ovarian cancers are included below as mechanistic comparators, not as interchangeable examples of established obesity-attributable risk. To distinguish epidemiological associations from experimentally demonstrated mechanisms, **Table 2** compares representative cancer-specific studies, experimental models, key mechanistic findings, and the limitations of obesity-related inference.

**Table 2:** Cancer-specific primary evidence for neural–immune–metabolic mechanisms and limits of obesity-related inference.

Cancer type	Obesity-related evidence	Representative study/model	Key mechanistic findings	Evidence limitations
Breast Cancer	Obesity-associated risk varies by menopausal status and tumor subtype (1).	Hoover et al., Nature 2025; neuronal–tumor experimental systems and metastatic breast cancer models (29).	Nerve-to-cancer mitochondrial transfer enhanced metabolic adaptation during metastatic progression.	Direct neural–metabolic mechanism; obesity-induced neural signaling was not tested.
Colorectal	Established association with excess adiposity (1).	Kobayashi et al., Cancer Discov 2025; CRC models, CAF experiments and human specimens (38).	NE–ADRB2 signaling in CAFs increased NGF and sympathetic innervation; tumor ADRA2A–YAP and NGF–PI3K/AKT supported growth.	Direct nerve–stroma feedback, not an experimentally established obesity→neural→CRC pathway.
Pancreatic (PDAC)	Excess adiposity is an established risk-related exposure (1).	Renz et al., Cancer Cell 2018; KC/KPC mice and observational PDAC cohorts (39).	ADRB2–NGF/BDNF feedforward loop increased nerve density; β-blockade plus gemcitabine improved survival in KPC mice.	Therapeutic benefit is preclinical; human β-blocker associations are observational.
Endometrial	Strong epidemiologic link with adiposity; hormonal and metabolic effects are relevant (1, 28).	Leptin-stimulated endometrial cancer cell experiments (28); no matched neural-intervention study identified here.	Leptin activated STAT3/ERK and promoted proliferation in an endometrial cell model.	Obesity-related signaling is supported; this experiment did not test a neural mediator.
Esophageal adenocarcinoma	Excess adiposity is associated with adenocarcinoma risk (1).	Human adiposity–cancer epidemiologic evidence (1); no adenocarcinoma-specific neural causal experiment established in this comparison.	A direct neural–immune mechanism linking obesity to this histologic subtype remains unverified.	Explicit evidence gap; do not extrapolate from unspecified esophageal cancer cell lines.
Hepatocellular carcinoma	Risk depends on metabolic liver disease, etiology and adiposity (1, 9).	Huan et al., Brain Behav Immun 2017; patient HCC tissue and DEN-induced rat models (40).	Sympathetic nerve input activated Kupffer-cell α1-AR signaling and IL-6/TGF-β; denervation or α1 blockade reduced experimental tumors.	Direct neural–immune mechanism in animals; obesity-specific mediation not demonstrated.
Prostate (comparator)	Not treated here as a uniformly obesity-attributable incidence cancer.	Zahalka et al., Science 2017; mouse prostate cancer and endothelial Adrb2/Cox10 perturbations (41).	Neural NE–endothelial ADRB2 altered oxidative phosphorylation and angiogenesis; Adrb2 deletion suppressed tumor growth.	Strong nerve–vascular–metabolic model; does not demonstrate obesity as the upstream exposure.
Ovarian (comparator)	Adiposity associations depend on histologic subtype; not assumed uniform.	Thaker et al., Nat Med 2006; orthotopic ovarian carcinoma mouse models (42).	Chronic stress increased catecholamines; tumor ADRB2–cAMP/PKA increased VEGF, MMP2/9, angiogenesis and tumor burden.	Stress exposure is not obesity; no established clinical survival benefit from neural targeting.

## 6 Conclusions

The relationship between obesity and cancer extends beyond adipose inflammation and metabolic dysfunction (43, 44). Emerging evidence supports a broader systems model in which obesity-associated inflammatory and metabolic stress perturbs central and peripheral neural homeostasis, while neural remodeling creates new routes of communication among cancer cells, immune cells, and stromal cells (45–48). Within this network, sympathetic and neuroendocrine signals, inflammatory pathways, and tumor metabolism can reinforce one another, potentially generating a neuroimmunometabolic environment favorable to tumor progression and treatment resistance.

The obesity–neuroinflammation–cancer axis should therefore be regarded as a testable mechanistic framework rather than a universal pathway. Its value lies in connecting obesity biology, neuroinflammation, cancer neuroscience, tumor immunology, and metabolism, while highlighting candidate points at which these systems may be therapeutically uncoupled. The next phase of the field should establish cancer-type-specific causal circuits, define reproducible neuroimmune phenotypes, and test biomarker-guided combination strategies.

This narrative Review also has inherent limitations. It integrates evidence from heterogeneous experimental models, cancer types, and research disciplines without a prespecified systematic-search or quantitative-synthesis protocol; consequently, the proposed framework should be interpreted as a hypothesis-generating synthesis rather than an estimate of effect size or certainty. Prospective mechanistic and clinical studies will be required to determine which components of the axis are causal, generalizable, and therapeutically actionable.
